# When anger dominates the mind: Increased motor corticospinal excitability in the face of threat

**DOI:** 10.1111/psyp.12685

**Published:** 2016-06-21

**Authors:** Ruud Hortensius, Beatrice de Gelder, Dennis J. L. G. Schutter

**Affiliations:** ^1^ Brain and Emotion Laboratory, Department of Cognitive Neuroscience, Faculty of Psychology and NeuroscienceMaastricht UniversityThe Netherlands; ^2^ Cognitive and Affective Neuroscience Laboratory, Department of Medical and Clinical Psychology, Tilburg School of Social and Behavioral SciencesTilburg UniversityTilburgThe Netherlands; ^3^ Department of Psychiatry and Mental Health, Faculty of Health SciencesUniversity of Cape TownCape TownSouth Africa; ^4^ Donders Institute of Brain, Cognition and Behaviour, Radboud University NijmegenNijmegenThe Netherlands

**Keywords:** Threat, Anger, Fear, Bodily expressions, TMS, Motor corticospinal excitability

## Abstract

Threat demands fast and adaptive reactions that are manifested at the physiological, behavioral, and phenomenological level and are responsive to the direction of threat and its severity for the individual. Here, we investigated the effects of threat directed toward or away from the observer on motor corticospinal excitability and explicit recognition. Sixteen healthy right‐handed volunteers completed a transcranial magnetic stimulation (TMS) task and a separate three‐alternative forced‐choice emotion recognition task. Single‐pulse TMS to the left primary motor cortex was applied to measure motor evoked potentials from the right abductor pollicis brevis in response to dynamic angry, fearful, and neutral bodily expressions with blurred faces directed toward or away from the observer. Results showed that motor corticospinal excitability increased independent of direction of anger compared with fear and neutral. In contrast, anger was better recognized when directed toward the observer compared with when directed away from the observer, while the opposite pattern was found for fear. The present results provide evidence for the differential effects of threat direction on explicit recognition and motor corticospinal excitability. In the face of threat, motor corticospinal excitability increases independently of the direction of anger, indicative of the importance of more automatic reactions to threat.

“Evolution created several coherently operating neural systems that help orchestrate and coordinate perceptual, behavioral, and physiological changes that promote survival in the face of danger” (Panksepp, 1998, p. 206).

In the human brain, both subcortical and cortical areas underlie defensive mechanisms when confronted with threat (de Gelder, Snyder, Greve, Gerard, & Hadjikhani, 2004; Mobbs et al., 2007; Panksepp, 1998; Pichon, de Gelder, & Grèzes, 2012). Adaptive reactions to threat depend on a balance between these areas (e.g., van Honk, Harmon‐Jones, Morgan, & Schutter, 2010). Emotional reactions to threat, including anger and fear, are influenced by several factors, such as personality and cognitive appraisal (Dill, Anderson, Anderson, & Deuser, 1997; Hall & Davidson, 1996; Wilkowski, Robinson, Gordon, & Troop‐Gordon, 2007). Furthermore, threatening signals are decoded and interpreted within a contextual setting (Kret & de Gelder, 2010; Righart & de Gelder, 2008a, 2008b; Sinke, Van den Stock, Goebel, & de Gelder, 2012; Van den Stock, Vandenbulcke, Sinke, & de Gelder, 2014).

Previous research mainly looked at the processing of threat signals without taking into account the observers' perspective. However, investigating threat signals independent of whether the threat is directed toward or away from the observer may introduce ambiguity of the threatening stimulus. For example, the fearful face may be interpreted in at least two ways, namely, as a consequence of a threat in the environment or as a consequence of an action of the observer. One way to take into account the perspective of the observer is the use of gaze direction (Hadjikhani, Hoge, Snyder, & de Gelder, 2008; Langton, Watt, & Bruce, 2000; N'Diaye, Sander, & Vuilleumier, 2009).

For faces expressing threat, gaze disentangles the relevance with respect to the observer. A fearful facial expression with averted gaze signals a possible imminent threat in the environment, similar to an angry facial expression with direct gaze that signals direct threat to the observer. In agreement, angry faces with direct gaze and fearful faces with averted gaze are recognized faster (Adams & Kleck, 2003), rated as more intense (Adams & Kleck, 2005; see also Hess, Adams, & Kleck, 2007; N'Diaye et al., 2009; Sander, Grandjean, Kaiser, Wehrle, & Scherer, 2007), and promote fast reactions to facial expressions (Soussignan et al., 2013). An fMRI study found increased activation to fearful facial expressions with averted compared to direct gaze, not only in brain areas important for stimulus detection, but also in action preparation (premotor and motor areas; Hadjikhani et al., 2008). Comparable results have been found when manipulating the relevance of angry bodily expressions (Grèzes, Adenis, Pouga, & Armony, 2013; Grèzes, Philip et al., 2013). Similar to a dynamic dual route perspective of affective perception (de Gelder, Hortensius, & Tamietto, 2012), Grèzes and colleagues showed that a first network consisting of the premotor area, inferior frontal gyrus, amygdala, and temporal pole is not necessarily modulated by personal relevance, but is particularly important for rapid detection and responses to threat (Grèzes, Adenis et al., 2013). The second network, consisting of the somatosensory cortices and the ventromedial prefrontal cortex, does depend on personal relevance and is suggested to code for somatic consequences of the emotional state in the observer and response selection. As the direction to and distance from the observer are important for emotional memory and the behavioral consequences (i.e., fight, flight, or freeze) of the perceived threat (Åhs, Dunsmoor, Zielinski, & LaBar, 2015; Blanchard & Blanchard, 1989), we aimed to extend previous findings by using the direction of the action as communicated by movement to investigate the effect of threat directed toward or away from the observer, on the level of physiology and explicit threat recognition.

To directly quantify the effect on motor corticospinal excitability levels when an individual is confronted with threat, we used single‐pulse transcranial magnetic stimulation (TMS). When applied to the primary motor cortex (M1), motor neurons can be excited by delivering a strong, brief magnetic pulse to the scalp, leading to a motor evoked potential (MEP) that indexes motor corticospinal excitability (Hallett, 2000). Early findings by Fadiga, Fogassi, Pavesi, and Rizzolatti (1995) showing that action observation increased motor corticospinal excitability were extended by a later study showing effects of self‐induced happiness and sadness on motor corticospinal excitability levels (Tormos, Cañete, Tarazona, Catalá, & Pascual‐Leone, 1997). Indeed, motor corticospinal excitability levels have successfully served as a proxy for emotion‐related action mechanisms in a variety of studies (Avenanti, Bueti, Galati, & Aglioti, 2005; Baumgartner, Willi, & Jäncke., 2007; Borgomaneri, Gazzola, & Avenanti, 2012; Coelho, Lipp, Marinovic, Wallis, & Riek, 2010; Coombes et al., 2009; Enticott et al., 2012; Giovannelli et al., 2013; Hajcak et al., 2007; Overeem, Reijntjes, Huyser, Lammers, & van Dijk, 2004; Schutter, Hofman, & van Honk, 2008; van Loon et al., 2010). Furthermore, Schutter et al. (2008) showed that fearful facial expressions selectively increase motor corticospinal excitability, suggesting increased action preparedness when confronted with threat (Hajcak et al., 2007).

In the present study, we used single‐pulse TMS to study the physiological consequence of threat directed toward or away from the observer. We showed participants dynamic video clips of social threat, with fear and anger as threat signals, and measured motor corticospinal excitability levels and explicit recognition. The goal of our study was to address the question of whether motor corticospinal excitability levels and explicit recognition were directly related to the direction of threat. We anticipated that anger directed toward the observer and fear directed away from the observer is better recognized than anger directed away from the observer and fear directed toward the observer. The central question was whether a similar effect is observed for motor corticospinal excitability levels. We expected that motor corticospinal excitability levels in response to threat are independent of the direction. That is, motor corticospinal excitability levels increase regardless of whether anger is directed toward the observer or away from the observer. A similar modulation of motor corticospinal excitability levels independent of direction would be expected for fearful expressions.

## Method

### Participants

Participants were recruited by advertisements around the Utrecht University campus and by means of word of mouth. While we did not conduct a formal power analysis, we decided to test at least 16 participants based on previous studies that used single‐pulse TMS to investigate emotion processing. For these studies, the mean number of participants reported is 15.08 with a standard deviation of 4.83, with a minimum of seven participants and a maximum of 24 participants, well suited to find medium to large effect sizes (Avenanti et al., 2005; Avenanti, Minio‐Paluello, Bufalari, & Aglioti, 2006, 2009; Avenanti, Minio‐Paluello, Sforza, & Aglioti, 2009; Baumgartner et al., 2007; Borgomaneri et al., 2012; Borgomaneri, Gazzola, & Avenanti, 2014, 2015; Borgomaneri, Vitale, & Avenanti, 2015; Borgomaneri, Vitale, Gazzola, & Avenanti, 2015; Coelho et al., 2010; Coombes et al., 2009; Enticott et al., 2012; Fadiga et al., 1995; Giovannelli et al., 2013; Hajcak et al., 2007; Overeem et al., 2004; Schutter et al., 2008; van Loon et al., 2010). Eighteen healthy right‐handed volunteers (14 women, four men), aged between 18 and 24 years, participated in exchange for course credits or payment. Participants had normal or corrected‐to‐normal vision, no contraindications for noninvasive brain stimulation (Keel, Smith, & Wassermann, 2001), or history of psychiatric or neurological disease. None of the participants were regular smokers or were on medications, except for women using oral contraceptives (*n* = 10). All participants received written and oral information prior to the study, but remained naïve about the aim of the study, and provided written informed consent. Stimulation parameters were in agreement with the International Federation of Clinical Neurophysiology safety guidelines (Rossi, Hallett, Rossini, Pascual‐Leone, Safety of TMS Consensus Group, 2009), and the study was approved by the medical ethics committee of University Medical Center Utrecht and Utrecht University, Utrecht, The Netherlands and carried out in accordance with the standards set by the Declaration of Helsinki.

### Stimuli

**Figure 1:**
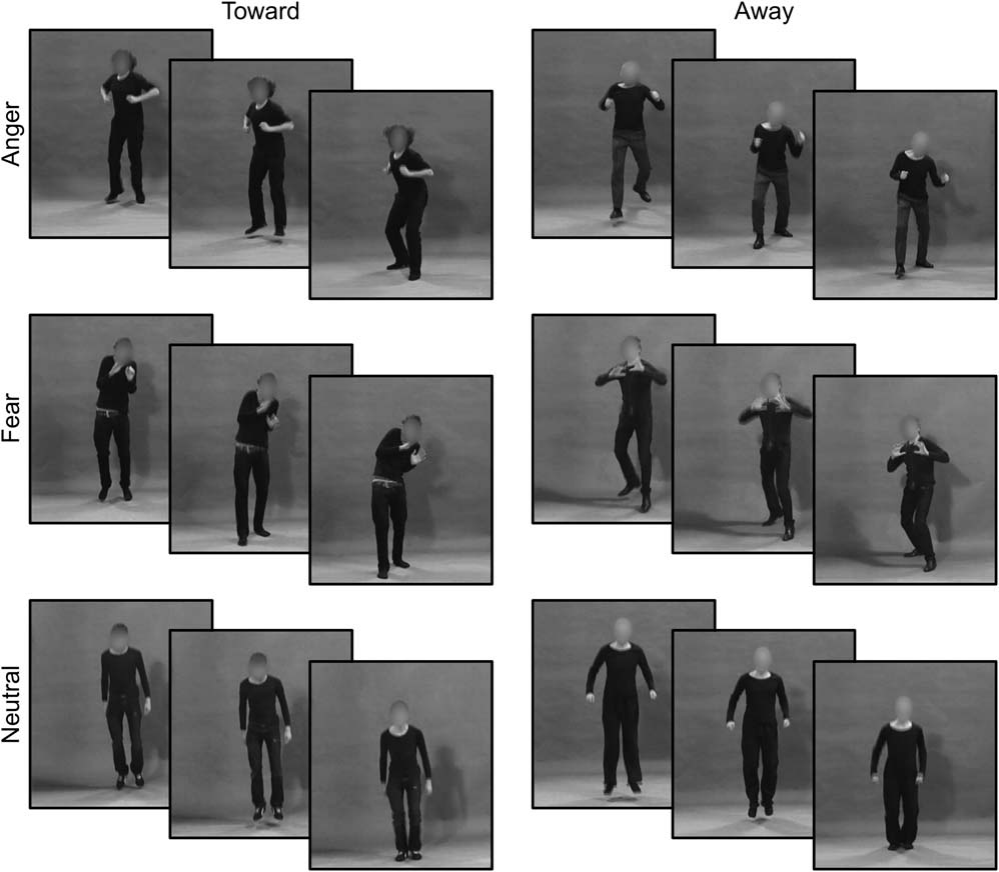
Example frames of the stimuli used.

Dynamic emotional expressions directed toward or away from the observer were recorded as part of the creation of a larger stimulus database containing facial and bodily expressions (see Kret, Pichon, Grèzes, & de Gelder, 2011). In order to achieve natural expressions of emotions during the recording, actors read short emotion‐inducing stories, were shown pictures of emotional scenes, and were coached throughout the recordings. Eight male actors expressed anger or fear toward or away from the observer by means of a forward or backward jump. Thus, we were able to create congruent and incongruent expressions of the emotion. In the congruent condition, anger is expressed toward the observer (forward jump), while fear is expressed away from the observer (backward jump). In the incongruent condition, anger is expressed away from the observer, and fear is expressed toward the observer. It is important to note that the perspective of the observer defines the direction of threat. Thus, the angry or fearful individual was moving toward, or away from, the observer by means of a jump. Threat was always directed at the observer as the actor had direct gaze and a frontal body orientation. Only the direction of the jump differed between the threat directed toward and away from the observer, as all other aspects were held constant. To allow for control of movement, a neutral expression was also included. For each actor and emotion, two different versions were recorded. Actors were dressed in black and filmed against a green background in a recording studio under controlled and standardized lighting conditions. Video clips (2‐s) were edited using Adobe After Effects CS5 (Adobe Systems Inc., San Jose, CA). Faces were masked with Gaussian mask in order to focus on information communicated by the body. Duration of the clip was reduced to 300 ms since previous studies found an increase in motor corticospinal tract excitability 300 ms after stimulus onset (Oliveri et al., 2003; Schutter et al., 2008). Figure 1 and the online supporting information video show examples of the stimuli used.

### Transcranial Magnetic Stimulation and Motor Evoked Potentials

A biphasic Neopulse magnetic brain stimulator (maximum output 4160 A peak/1750 VAC peak) with a modified 8‐shaped iron core coil (Neopulse, Atlanta, GA) was used for stimulation over the left M1.

Motor evoked potentials were recorded with active Ag‐AgCl electrodes (11 × 17 mm) using an ActiveTwo system (BioSemi, Amsterdam, The Netherlands) from the right abductor pollicis brevis (APB) in a belly‐tendon montage with the active electrode placed at the muscle belly of the right APB and the reference electrode located at the proximal phalanx of the thumb. The ground electrode was attached to the wrist. Sampling rate was set at 2048 Hz, and the signal was offline high‐pass filtered (3 dB cutoff frequency: 20 Hz roll‐off: 24 dB/octave). MEP from the APB to single‐pulse TMS is a robust and reliable proxy for motor corticospinal excitability (Baumgartner et al., 2007; Borgomaneri et al., 2012; Borgomaneri, Gazzola, & Avenanti, 2014; Hajcak et al., 2007; Schutter et al., 2008), with distinct thumb contractions and lower motor thresholds and steeper MEP recruitment compared to other muscles (Hajcak et al., 2007).

### Procedure

**Figure 2:**
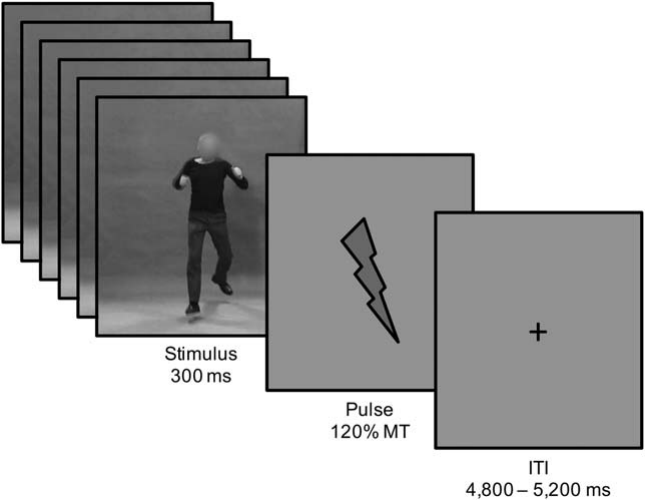
Transcranial magnetic stimulation (TMS) task.

After explanation of the procedure by the experimenter, the participants provided written informed consent and answered several standard questions on present physical and mental well‐being (including hours of sleep and caffeine and alcohol intake in the last 24 h, and current emotional state) as an additional check for exclusion criteria. Next, participants were seated in a comfortable dentist chair with their arms placed on the upper legs with the palm of the hand facing upward. Electromyogram (EMG) electrodes were attached, and the resting motor threshold of the left hemisphere was assessed (mean ± *SD* percentage of maximum output: 49.21 ± 7.04%), using the standardized visual thumb movement procedure (Schutter & van Honk, 2006). A passive viewing task was used, and participants were instructed to relax their body, not focus on their hands, and fixate on the fixation cross shown continuously during the task. Single‐pulse TMS over left M1 at an intensity of 120% motor threshold was applied 300 ms after stimulus onset. After completion of the TMS procedure, participants indicated the emotion (fear, anger, or neutral) of the presented stimulus in a separate three‐alternative forced‐choice task. Stimuli (16 per condition) were presented in random order with a fixation cross (Figure 2; TMS: 4,800–5,200 ms; emotion recognition: 1,000–1,500 ms) in between. Upon completion, participants were debriefed and received payment.

### Data Reduction and Analysis

Data of two participants were removed due to noisy EMG signals and excessive muscle artifacts.

MEP was quantified as the peak‐to‐peak amplitude (> 50 μV) of the maximal EMG response. Every trial was visually inspected and was done blind to the stimulus condition (Avenanti et al., 2005; Coelho et al., 2010; Hajcak et al., 2007; van Loon et al., 2010). Trials containing background EMG two *SD* from the mean (Borgomaneri et al., 2012; Borgomaneri, Vitale, Gazzola, & Avenanti, 2015) and MEPs < 50 μV or outside of the expected time window were removed. Mean ± *SD* of included trials per condition across participants was 14.07 ± 2.37. The number of trials removed did not differ between conditions (*p*s ≥ .59). As the data were significantly nonnormal distributed, *D*(16) = 0.26, *p* = .004, and to reduce interindividual variability, MEPs were transformed into *z* scores based on individual mean and standard deviation (cf. Burle, Bonnet, Vidal, Possamaï, & Hasbroucq, 2002; Fadiga et al., 1995; Giovannelli et al., 2013; van Loon et al., 2010). In addition, mean rectified baseline EMG activity was epoched from 1,010 ms to 10 ms prior to the TMS pulse in order to examine the possible effect of baseline EMG activity on the MEP (Devanne, Lavoie, & Capaday, 1997; Orban de Xivry, Ahmadi‐Pajouh, Harran, Salimpour, & Shadmehr, 2013). To check if timing of the TMS pulse (0.19 Hz, mean ± *SD* interval between pulses was 5,302 ± 2.03 ms) influenced motor corticospinal excitability, we contrasted the first and second half of the trials. No significant increase or decrease in MEP amplitude was observed, *t*(15) = .38, *p* = .71. Similarly, no effect was found for baseline EMG activity, *t*(15) = .85, *p =* .41. For the emotion recognition data, we calculated the recognition accuracy (percentage correct) for each emotion as a function of direction. In addition, for each emotion, an incongruence effect was calculated by subtracting recognition accuracy of expressions directed away from the observer from recognition accuracy of expression directed toward the observer. A positive value indicated better recognition when the emotion is directed toward the observer, whereas a negative value indicated better recognition when the emotion is directed away from the observer.

A repeated measures analysis of variance (ANOVA) with direction (2) and emotion (3) as within‐subject factors was performed for both the TMS and emotion recognition data. Paired‐sample *t* tests were performed for post hoc testing. The alpha level of significance was set at 0.05 (two‐tailed) throughout.

## Results

### Motor Corticospinal Excitability

**Figure 3:**
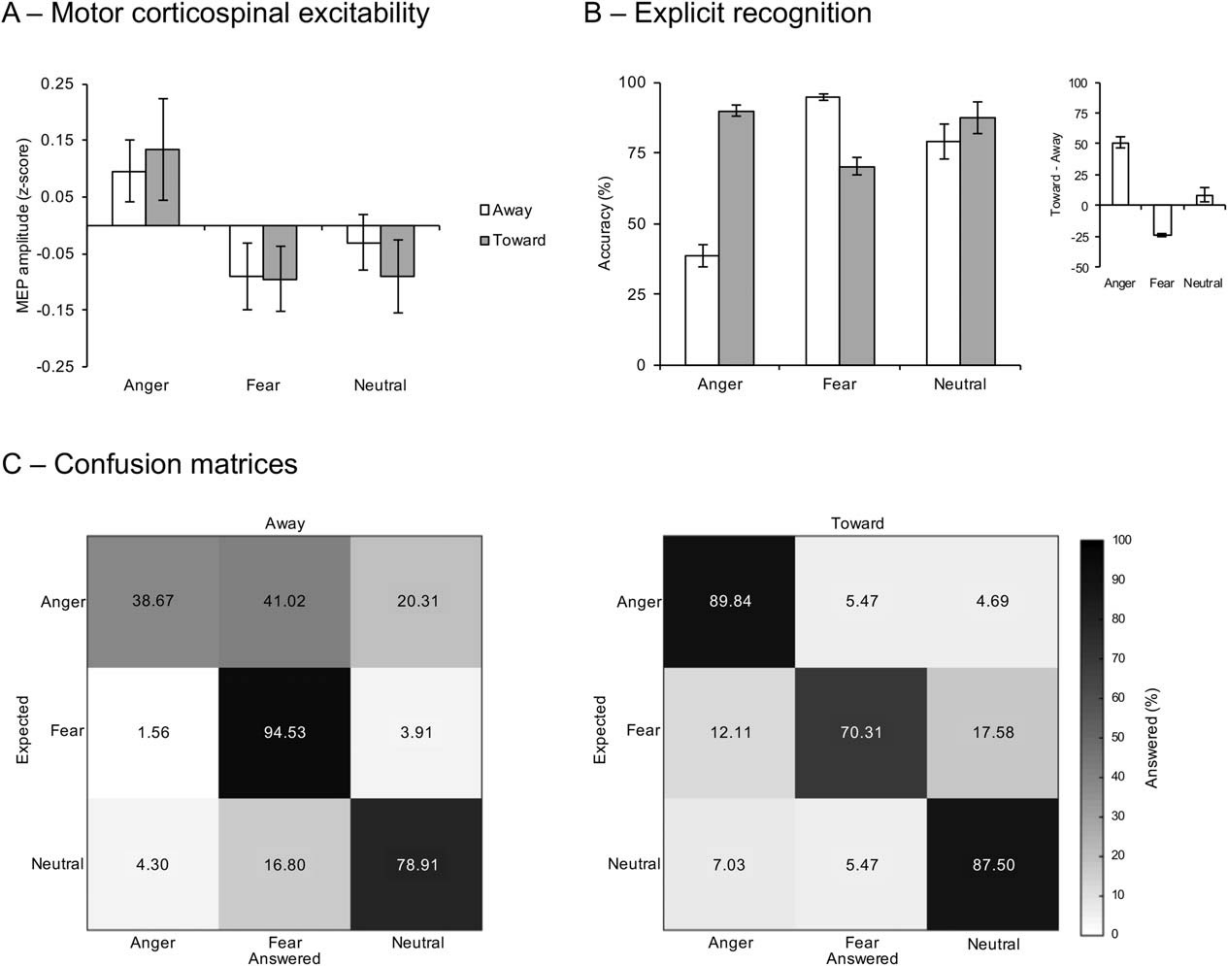
The effect of direction of threat on motor corticospinal excitability levels and explicit recognition accuracy. MEP amplitude did increase for anger independent of direction (A). Recognition accuracy was higher for angry expressions directed toward the observer and fear expressions directed away from the observer (B). Inset shows the incongruence effect. Anger directed away was confused with fear, while no clear confusion was observed for fear directed toward the observer (C).

Stimulation was well tolerated by all subjects, and no side effects were reported. No significant main effect of direction was observed, *F*(1,15) = 0.04, *p* = .85, whereas a significant main effect was found of emotion *F*(2,30) = 3.99, *p* = .03, η_p_^2^ = 0.21 (Figure 3A). The two‐way interaction between direction and emotion was not significant, *F*(2,30) = 0.30, *p* = .74. Post hoc tests show that MEP amplitude was increased independent of direction for anger (mean ± *SEM z*‐transformed MEP amplitude: 0.11 ± 0.05) compared with both fear (−0.09 ± 0.05) and neutral (−0.06 ± 0.04), *t*(15) = 2.52, *p* = .02, *d* = 0.63 and *t*(15) = 2.41, *p* = .03, *d* = 0.60, respectively. No difference was observed between fear and neutral expressions, *t*(15) = 0.36, *p* = .72. MEP amplitude differed only from zero for anger, *t*(15) = 2.33, *p* = .03, *d* = 0.58, and not for fear or neutral, *t*(15) = −2.04, *p* = .06 and *t*(15) = −1.49, *p* = .16, respectively.

These effects could not be explained by condition‐specific effects on baseline EMG activity, since no main effect of direction, *F*(1,15) = 2.30, *p* = .15, emotion, *F*(2,30) = 1.81, *p* = .19, or interaction between direction and emotion was found, *F*(2,30) = 2.22, *p* = .15. No significant correlations were found between baseline EMG activity and MEP amplitude within the different conditions, *p*s ≥ .22. Similar results were obtained after controlling for percentage of trials removed (centered), *F*(2,28) = 5.03, *p* = .01, η_p_^2^ = 0.26, with an increase in MEP amplitude for anger independent of direction compared with both fear, *p* = .008, and neutral, *p* = .03.

### Explicit Recognition

A main effect of direction, *F*(1,15) = 34.13, *p* < .001, η_p_^2^ = 0.70, and emotion, *F*(2,30) = 11.51, *p* =.006, η_p_^2^ = 0.34, was found. In addition, an interaction between direction and emotion was observed, *F*(2,30) = 127.12, *p* < .001, η_p_^2^ = 0.89 (Figure 3B). Recognition accuracy of angry expressions was higher when directed toward the observer (mean ± *SEM* percentage correct: 89.84 ± 1.88%) compared with away from the observer (38.67 ± 4.16%), *t*(15) = 11.56, *p* < .001, *d* = 2.89. The same pattern was observed for neutral expressions (toward: 87.50 ± 5.71%, and away: 78.91 ± 6.09%), *t*(15) = 3.67, *p* = .002, *d* = 0.92, whereas the opposite was found for fearful expressions (toward: 70.31 ± 3.20%, and away: 94.53 ± 1.12%), *t*(15) = 7.77, *p* < .001, *d* = 1.94. The incongruence effect was most profound for angry expressions (mean ± *SEM* toward–away difference: 51.17 ± 4.43) compared with fearful (24.22 ± 3.12; reversed), *t*(15) = 4.83, *p* < .001, *d* = 1.21, and neutral expressions (8.59 ± 2.34), *t*(15) = 9.04, *p* < .001, *d* = 2.26. The incongruence effect for fearful expression was significantly higher compared with neutral expressions, *t*(15) = 4.39, *p* = .001, *d* = 1.10.

Assessment of response patterns in the incongruent conditions showed that, when directed away from the observer, anger (mean ± *SEM* percentage of answers: 38.67 ± 4.16%) was more likely to be confused with fear (41.02 ± 4.60%) than with neutral (20.31 ± 3.05), *t*(15) = 3.14, *p* = .007, *d* = 0.78 (Figure 3C). No confusion was observed for fear directed toward the observer (70.31 ± 3.20%), with no difference between percentage of anger (12.11 ± 2.77%) and neutral responses (17.59 ± 3.39%), *t*(15) = −1.03, *p* =.32.

A generalized linear mixed model with direction and emotion as fixed effects and participant as random effect with a binomial distribution including pairwise contrasts revealed similar results. While no significant main effect of direction was found, *F*(1,90) = 2.98, *p* = .09, a significant main effect of emotion, *F*(2,90) = 14.11, *p* < .001, as well as a significant interaction between direction and emotion, *F*(2,90) = 74.50, *p* < .001, was observed. Pairwise contrasts showed that angry expressions were recognized better when directed toward the observer compared with away from the observer, *t*(90) = 11.20, *p* < .001. Again, the opposite result was found for fearful expressions, *t*(90) = 7.14, *p* < .001. Recognition accuracy for fearful expressions away from the observer was higher compared with fearful expressions toward the observer.^1^

To check the dependence of motor corticospinal excitability levels on explicit recognition, we performed two control analyses. First, we tested if recognition accuracy influenced the results. For this analysis, only the TMS trials were analyzed that contained stimuli that were correctly recognized in the explicit recognition task. No main effect of direction, *F*(1,14) = 1.75, *p* = .21, emotion, *F*(2,28) = 2.41, *p* =.11, or interaction between direction and emotion, *F*(2,28) = 0.11, *p =* .90, was found. Second, we used the subjective classification of the expressions in the explicit recognition task to classify the TMS trials. For example, if a participant classified an “angry expressions directed away” as fear, the expression was classified as part of the latter category in the TMS task. Again, no main effect of direction, *F*(1,14) = 0.30, *p* = .60, emotion, *F*(2,28) = 2.93, *p* =.07, or interaction between direction and emotion, *F*(2,28) = 0.10, *p =* .90, was found. One participant was removed from the analysis because the participant consistently classified neutral expression directed away from the observer as fear (69%) or anger (31%).

In a separate behavioral study (*n* = 27), we replicated the effects on explicit recognition. An interaction between direction and emotion was observed, *F*(2,52) = 139.07, *p* < .001, η_p_^2^ = 0.84, with recognition of angry (toward: 84.49 ± 2.17%, away: 43.06 ± 3.54%, *t*(26) = 11.78, *p* < .001, *d* = 2.27) and fearful expressions (toward: 69.91 ± 2.16%, and away: 92.36 ± 1.98%, *t*(26) = 9.31, *p* < .001, *d* = 1.79) showing opposite results. Anger directed away from the observer was more likely to be confused with fear (35.19 ± 3.17%) than with neutral (21.76 ± 2.66%), *t*(26) = 2.88, *p* = .008, *d* = 0.56. In this sample, fear directed toward the observer was more likely to be confused with neutral (20.83 ± 2.03%) than with anger (9.26 ± 1.81%), *t*(26) = 3.64, *p* =.001, *d* = 0.70. Similar results were obtained when using a generalized linear mixed model, *F*(2,156) = 58.93, *p* < .001, with pairwise contrasts for anger, *t*(156) = 9.94, *p* < .001, and fear, *t*(156) = 8.24, *p* < .001.

## Discussion

The goal of the present study was to evaluate the influence of direction of threat from the perspective of the observer using measures of motor corticospinal excitability and explicit recognition. Results showed a dissociation between motor corticospinal excitability and explicit recognition. Interestingly, motor corticospinal excitability levels increased independent of direction of anger. However, explicit recognition results showed an incongruence effect for fearful and angry expressions. Recognition accuracy was higher for anger directed toward the observer compared to anger directed away from the observer, while the opposite pattern was found for fearful expressions.

Our results concur with evolutionary accounts on emotion (Darwin, 1872/2009) and highlight the emotion‐action link (Frijda, 1986). The influence of threat can be observed at three interrelated levels in the organism: perception, behavior, and physiology (Panksepp, 1998). Effective threat processing depends on the ability to perceive threat as such, and the consequent physiological changes that eventually would lead to adaptive behavior. Threats in the environment lead to a cascade of reactions in the observer, preparing possible behavioral consequences (Frijda, 2010), such as startle responses (Lang, Bradley, & Cuthbert, 1990), fast facial reactions (Dimberg & Thunberg, 1998), and changes in heart rate (Graham & Clifton, 1966). What mechanism and neural network may underlie these initial reactions?

The dynamic dual‐route perspective of affective perception suggests that one route underlies early emotion processing that results in reflexive action, while a cortical‐based network underlies conscious recognition and voluntary actions (de Gelder et al., 2012). Importantly, a network consisting of the periaqueductal gray, hypothalamus, amygdala, the premotor cortex, and presupplementary motor area mediates behavioral reactions of the individual when confronted with a threatening situation (de Gelder et al., 2004; Grèzes, Adenis, Pouga, & Armony, 2013; Grèzes, Pichon, & de Gelder, 2007; Grosbras & Paus, 2006; Pichon et al., 2012; Pichon, de Gelder, & Grèzes, 2008, 2009). The confrontation with a conspecific displaying anger could directly activate a reflexive mechanism in the observer. Similar to that in monkeys (e.g., Avendaño, Price, & Amaral, 1983) a direct amygdala‐motor cortex network has recently been found in humans (Grèzes, Valabrègue, Gholipour, & Chevallier, 2014). This network would allow for relatively direct activation of the motor system without top‐down influences in the face of threat. This view is in agreement with the activation of this network independent of relevance of (Grèzes, Adenis, Pouga, & Armony, 2013) and attention to (Pichon et al., 2012) angry bodily expressions.

Preparation for defensive reactions not only needs to be relatively independent of attention and other cognitive processes, but also needs to be early and fast as well. Based on previous research (Oliveri et al., 2003; Schutter et al., 2008), we stimulated the motor cortex 300 ms poststimulus onset and found a selective increase for angry bodily expressions. Interestingly, Borgomaneri, Gazzola, & Avenanti (2014) showed that at 150‐ms poststimulus onset, motor corticospinal excitability increased only for stimuli negative in valence, while at 300‐ms poststimulus onset, it increased for both stimuli negative and positive in valence (Baumgartner et al., 2007; see also Borgomaneri et al., 2012; Borgomaneri, Vitale et al., 2015; Coombes et al., 2009; Hajcak et al., 2007). In contrast, Schutter and colleagues (2008) found that fearful, but not happy or neutral faces increased motor corticospinal excitability as measured at 300 ms after stimulus onset. So far, the temporal dynamics of the influence of emotional signals on motor corticospinal excitability remain elusive.

The observation that fearful bodily expressions toward or away from the observer did not affect motor corticospinal excitability levels is not necessarily in contradiction with a previous study showing a selective increase for static fearful facial expressions (Schutter et al., 2008). Next to differences in terms of communicative value and immediacy between faces and bodies (de Gelder, 2009), static versus dynamic emotional signals (Grèzes et al., 2007), proximate versus distal threat (Mobbs et al., 2007), and contextual differences in relevance and threat value (Mobbs et al., 2010) could explain the difference in results. In the present study, angry bodily expressions could have had the highest relevance to the participant and the highest threat value compared to fearful and neutral expressions. In the previous study with facial expression by Schutter and colleagues (2008) and in other studies using bodily expressions (Borgomaneri et al., 2012; Borgomaneri, Gazzola, & Avenanti, 2015; Borgomaneri, Vitale, & Avenanti, 2015; Borgomaneri, Vitale et al., 2015), fearful faces and postures had the highest relevance and threat value as compared to happy and neutral faces and postures. In the current study, we found that anger demonstrates the most pronounced effects. To counteract potential and unwanted effects of relevance and threat value, future studies should consider which emotional signals to include and compare, for example, by directly comparing threat signals such as fear and anger (Pichon et al., 2009, 2012). Interestingly, motor corticospinal excitability levels in response to fear were, while not significant, lower compared to angry and neutral signals. A recent study found a reduction in motor corticospinal excitability for fearful compared to happy and neutral bodily expressions 70–90 ms after stimulus onset (Borgomaneri, Vitale, & Avenanti, 2015). This finding is complemented by another TMS study that reported reduced facilitation of excitability levels within the motor cortex 100–150 ms after the presentation of a fearful bodily expression. While we stimulated 300 ms poststimulus onset, our results might point to an, albeit extended, freezelike process in response to expressions of fear.

An additional question is at what moment in time information of direction, relevance, and other contextual factors are combined. Early contextual effects (115–160 ms poststimulus onset) on the processing of emotion signals have been reported (Meeren, van Heijnsbergen, & de Gelder, 2005; Righart & de Gelder, 2008a). Interestingly, a combined EEG and fMRI study showed that, while processing in the amygdala of emotional content was independent of gaze and gesture, these factors are integrated at the level of the premotor cortex already 200 ms after stimulus onset (Conty, Dezecache, Hugueville, & Grèzes, 2012). In contrast, our results show that direction of anger is not affecting motor corticospinal excitability when stimulating at 300 ms poststimulus onset. In fact, the dynamic dual‐route model can explain this difference. Angry bodily expressions trigger activation of the first network, which is independent of direction, and result in activation of preparatory processes. It is important to note that these two networks do not necessarily have to be exclusive in terms of brain regions. The crucial distinction is that in one network contextual information is taken into account, while in the other network it is not. The present findings of increased motor corticospinal excitability, even if the angry person is jumping away from the observer, might also reflect aberrant activation of preparatory responses. It is possible that top‐down influences might counteract this initial process. These questions warrant further testing by probing the primary motor cortex at different time points.

The pattern of results found for explicit emotion recognition suggests that activation in the second network could underlie these results, as explicit recognition presumably uses different processing resources than the reactive aspect (e.g., de Gelder et al., 2012). Explicit processing may tap into more cognitive‐related processes. It takes the form of categorization (e.g., Is it an angry, fearful, or neutral person? How angry is the person?) instead of a binary response (e.g., Threat or no‐threat? Is this something that I need to act upon?). In line with previous modulation by relevance (Grèzes, Adenis, Pouga, & Armony, 2013), activation in regions of this network reflects the direction of threat. During explicit recognition and categorization of bodily expressions, direction of movement in reference to the observer is taken into account. The explicit recognition results are in line with a prototypical, but context‐dependent, distinction between approach and avoidance tendencies and anger and fear. From the perspective of the individual expressing the behavior, anger can be viewed as a manifestation of approach‐related behaviors, while fear can be viewed as a manifestation of avoidance‐related behaviors (Carver & Harmon‐Jones, 2009; Harmon‐Jones, 2003; Krieglmeyer & Deutsch, 2013; Wilkowski & Meier, 2010). This division might also be apparent at the perceptual level. Participants perceiving the emotional signal might be more inclined to respond with the label *fear* if an emotional movement is directed away from them and the label *anger* if the emotional movement is directed toward them. Indeed, categorization of angry facial expression is facilitated when accompanied by an approach movement (Adams, Ambady, Macrae, & Kleck, 2006), and approach‐related movements are faster for angry facial expressions (Wilkowski & Meier, 2010). Importantly, as suggested by the present experiment, these effects are dependent on context. For example, only when approach was linked to aggression did anger enhance approach movements (Krieglmeyer & Deutsch, 2013).

Factors that may mediate the processing of threat in contextual settings are personality and other inter‐individual differences. For example, violent offenders are more influenced by an irrelevant angry bodily expression when recognizing happy faces (Kret & de Gelder, 2013). Interestingly, people with a history of exposure to violent crimes compared to people with no history showed increased reaction times to threat directed toward them (Fernandes et al., 2013). Incorporating the perceptual and personality domain, a recent TMS study showed that interhemispheric connectivity was related to an attentional bias to angry facial expressions and to an aggressive personality style (Hofman & Schutter, 2009). As effects of personality on motor corticospinal excitability levels have also been reported (Avenanti, Minio‐Paluello, Bufalari, & Aglioti, 2009; Liuzza, Candidi, Sforza, & Aglioti, 2015; Wassermann, Greenberg, Nguyen, & Murphy, 2001), future studies may incorporate measures of aggression‐ and/or anxiety‐related traits in the study of perception and interpretation of threat and the occurrence of defensive and/or aggressive behavior.

An aspect that needs further consideration is the stimuli that were used in the present study. Here, we focused on the direction of threat as communicated directly by the movement of the person toward or away from the observer. We reduced the visibility of facial expressions by blurring the faces of the actors (Borgomaneri, Vitale, & Avenanti, 2015; Borgomaneri, Vitale, Gazzola, & Avenanti, 2015; de Gelder et al., 2004; Hortensius & de Gelder, 2014; Huis In 't Veld & de Gelder, 2015; Kret et al., 2011; Sinke, Sorger, Goebel, & de Gelder, 2010). This was done to maintain the focus of the participant on the main aspect of stimulus and away from facial information extraction, identity recognition, and other nonrelevant processes, Of course, facial and bodily expressions are heavily intertwined. For example, bodily expressions influence emotion and identity recognition and memory of faces (Aviezer et al., 2008; Aviezer, Trope, & Todorov, 2012; Van den Stock & de Gelder, 2012, 2014). The question arises whether similar results would be obtained if facial information were included. Would motor corticospinal excitability levels still increase for anger independent of the direction if the face of the aggressor was visible? How do facial expression and identity recognition influence the perception of bodily expressions? Are some emotions biased toward expression by face or body? Some studies suggest that bodily compared to facial expressions of anger are more relevant and salient to the observer (Hortensius, van Honk, de Gelder, & Terburg, 2014; Zhan, Hortensius, & de Gelder, 2015). It is possible that, if fear is signaled by the face in isolation or together with the body, motor corticospinal excitability levels might increase. Future studies may want to include bodily expressions with faces visible as well as face‐body compounds to systemically test its effects on motor corticospinal excitability levels and explicit recognition.

Finally, responses to TMS vary greatly among individuals (Wassermann, 2002). Besides an adequate sample size, several other methodological precautions should be made in order to achieve reliable measurements and subsequent results (Chipchase et al., 2012). First, we recorded a large and sufficient number of MEPs to achieve high within‐session reliability (Goldsworthy, Hordacre, & Ridding, 2016). Second, we controlled for prepulse activation of the target muscle (Darling, Wolf, & Butler, 2006; Devanne et al., 1997; Orban de Xivry et al., 2013). Third, besides well‐known criteria for contraindication (e.g., neurological and psychiatric disorders), we checked for intra‐ and interpersonal factors that influence excitability levels, that is, intake of caffeine (Cerqueira, de Mendonça, Minez, Dias, & de Carvalho, 2006), alcohol (Conte et al., 2008) and other psychoactive substances (Paulus et al., 2008), hours of sleep (Huber et al., 2013), and physical activity (Cirillo, Lavender, Ridding, & Semmler, 2009). Participants were asked to refrain from alcohol and caffeine intake and excessive physical activity 24 h prior to the experiment. In our opinion, these methodological considerations, together with the current sample size, have provided reliable results.

In conclusion, the present study shows that the direction of threat influences motor corticospinal excitability and explicit recognition differently. Importantly, motor corticospinal excitability increased independent of direction of anger, while explicit recognition was directly related to the direction of the emotional signal. This suggests that, in the face of threat, a rapid mechanism is activated that is independent of explicit recognition.

## Supporting information


**Video**: Examples of the stimuli.

## Footnote

1 While we found that neutral expressions directed toward the observer were better recognized compared with neutral expressions directed away from the observer, we do not discuss this finding further. The mean difference ± *SEM* in trials correct for neutral expressions was 1.38 ± 0.38 trials, compared to 8.19 ± 0.71 and 3.88 ± 0.5 for angry and fearful expressions, respectively. That is, on average, participants had 14 ± 0.91 out of 16 trials correct for neutral expressions directed toward the observer, while 12.63 ± 0.97 out of 16 trials correct for neutral expressions directed away from the observer. While this might be a consequence of enhanced processing of signals directed toward the observer, it is a very small effect. In addition, the difference in recognition accuracy between neutral expressions directed toward the observer and away from the observer was not significant in the generalized linear mixed model, *t*(90) = 1.03, *p* = .31.
